# SA2E: spatial-aware auto-encoder for cell type deconvolution of spatial transcriptomics data

**DOI:** 10.1093/bioinformatics/btag133

**Published:** 2026-03-20

**Authors:** Yaxiong Ma, Zengfa Dou, Yuhong Zha, Xiaoke Ma

**Affiliations:** School of Computer Science and Technology, Xidian University, Xi’an, Shaanxi 710071, China; School of Computer and Information Science, Qinghai Institute of Technology, Xining, Qinghai 810003, China; School of Computer Science and Technology, Xidian University, Xi’an, Shaanxi 710071, China; School of Computer Science and Technology, Xidian University, Xi’an, Shaanxi 710071, China

## Abstract

**Motivation:**

Spatial transcriptomics (ST) technologies measure gene expression together with spatial locations, but each spot typically contains a mixture of cell types, posing a challenge for downstream analysis. Cell-type deconvolution aims to infer spot-wise cell-type proportions by integrating single-cell RNA-seq (scRNA-seq) and ST data. Many existing methods construct cell-type signatures from predefined marker genes, which can limit performance when marker information is incomplete or unavailable.

**Results:**

To address this limitation, we propose a spatial-aware auto-encoder framework (SA2E) for cell-type deconvolution without requiring predefined cell-type biomarkers. SA2E learns latent spot representations using a spatially regularized auto-encoder that preserves the local topology of the spot spatial graph. Based on these representations, SA2E learns cell-type signatures by enforcing them to reconstruct ST expression. In our framework, simulated ST data with known proportions are used for supervised pretraining, while real ST data are optimized using the reconstruction objective. Extensive experiments on simulated and real ST datasets demonstrate that SA2E outperforms state-of-the-art deconvolution baselines.

**Availability and implementation:**

The code of SA2E is available at Github (https://github.com/xkmaxidian/SA2E) and Zenodo (DOI: 10.5281/zenodo.18765467).

## 1 Introduction

Cells execute their biological functions at the appropriate spatial location of tissues and organs in terms of cell populations (cell types) (Schürch *et al.* 2020). For instance, distinct neuronal and glial cell populations form layered or region-specific architectures of brains, which are typical biomarkers for complex micro-environments of tumors. High-throughput single-cell and single-nucleus RNA sequencing (sc/snRNA-seq) technology profiles genome-wide expression of cells, offering great opportunities for the identification of cell types, cell states and developmental trajectories (Tang *et al.* 2009, Macosko *et al.* 2015, Zheng *et al.* 2017). However, sc/snRNA-seq is also criticized for failing to fully characterize their functions since it loses spatial information of cells. Fortunately, spatial transcriptomics (ST) technologies address this limitation by jointly measuring transcription and spatial location of spots of tissues with various platforms (Ståhl *et al.* 2016, Longo *et al.* 2021), including image-based platforms [MERFISH (Chen *et al.* 2015), seqFISH (Moffitt *et al.* 2018) and osmFISH (Codeluppi *et al.* 2018)] and sequencing-based platforms [10× Visium (Ståhl *et al.* 2016) and Slide-seq (Rodriques *et al.* 2019)]. Notice that the image-based platforms increase resolution of spots by sacrificing genome coverage, whereas sequencing-based ones enhance genome coverage by sacrificing resolution.

Therefore, each spot of ST data generated by sequencing-based platforms corresponds to a mixture of cell types, and estimating the underlying composition of cell types (also called cell type deconvolution) is essential for downstream analyses, including the identification of spatial domains, micro-environment and spatially informative biomarkers (Ma and Zhou 2022). In other words, cell type deconvolution bridges gap between genome coverage and resolution. Thus, two critical tasks associated with cell type deconvolution, i.e. how to construct signatures of cell types as references, and how to estimate abundance of cell types for each spot. Current algorithms avoid the former task by assuming cell types are given and only focuses on designing algorithms to calculate abundance of cell types for each spots with various strategies.

According to the strategies for the estimation of abundance of cell types, current methods are divided into two categories, i.e. independent- and integration-based algorithms, where the former directly infer abundance of cell types, and the latter utilizes additional information to leverage the estimation of abundance of cell types. Representative independent-based algorithms include RCTD (Cable *et al.* 2022), Stereoscope (Andersson *et al.* 2020), SpatialDWLS (Dong and Yuan 2021), SPOTlight (Elosua-Bayes *et al.* 2021), and Cell2location (Kleshchevnikov *et al.* 2022), which differs greatly in terms of strategies for estimating mixtures of cell types. For example, Cell2location (Kleshchevnikov *et al.* 2022) decomposes mRNA counts of ST data using these reference signatures, and adopts a probabilistic model to estimate the relative and absolute abundance of cell types for each spot. RCTD (Cable *et al.* 2022) employs supervised learning for the decomposition of cell types, whereas SpatialDWLS (Dong and Yuan 2021) makes use of a linear model to estimate the composition of cell types. PanoSpace (He *et al.* 2026) integrates ST, histology, and matched scRNA-seq for single-cell-level spatial reconstruction. OmicsTweezer (Yang *et al.* 2025) is a deep learning-based deconvolution model designed for cross-platform, multimodal robustness. These algorithms significantly facilitate down-stream analysis by exploiting mixtures of cell types of ST data. However, these algorithms have limitations for many scenarios, such as heterogeneity of distributions, sizes and abundance of cell types, requiring additional information to fully characterize mixtures of cell types.

To address this limitation, many algorithms are developed by exploiting additional information of ST and signatures. The critical technique of these algorithms involves how to select and integrate additional information, such as the cell states, topic compositions, spatial auto-correlation and topological structure of graphs. Representative algorithms include CARD (Ma and Zhou 2022), DSTG (Song and Su 2021), STRIDE (Sun *et al.* 2022), DestVI (Lopez *et al.* 2022), MACD (Huang *et al.* 2024), eMCI (Hong *et al.* 2024), stGNN (Zhu *et al.* 2025), and jMF2D (Zha *et al.* 2025). For example, CARD (Ma and Zhou 2022) demonstrates that spatial information of cell type significantly improves performance of deconvolution since cell types execute their functions at specific locations of tissues. STRIDE (Sun *et al.* 2022) exploits semantic topics of signatures of cell types from scRNA-seq data, thereby enhancing specificity of cell types. stGNN (Zhu *et al.* 2025) and jMF2D (Zha *et al.* 2025) leverage topological structure of graphs to facilitate decomposition of cell types with different strategies, where the former only explore graph of spots, and the latter exploits multi-layer networks for transcription and spatial information. These algorithms dramatically improve performance of algorithms for cell type deconvolution, proving that integrating ST and complimentary information is promising.

Even though great efforts have been devoted to cell type deconvolution, constructing informative cell-type signatures remains a key challenge in spatial transcriptomics. Many existing methods build signatures by relying on predefined cell-type biomarkers, which are often obtained from scRNA-seq references or curated marker gene sets. This marker-dependent strategy has two major limitations. First, its applicability is constrained. Specifically, when reliable cell-type biomarkers are unavailable, these methods become difficult to apply due to the lack of informative signatures. Second, when the available biomarkers are incomplete or not sufficiently representative, deconvolution performance can substantially degrade because the corresponding cell types are poorly characterized.

Recently, reference-free approaches such as STdeconvolve (Miller *et al.* 2022) have made important progress by removing the requirement for scRNA-seq references, e.g. by using an LDA-based model to infer latent components directly from spot-level expression profiles. Nevertheless, these latent components are not explicitly learned as reconstruction-consistent cell-type signatures, and spatial neighborhood information is not directly integrated to preserve local spatial topology during feature learning.

**Figure 1:**
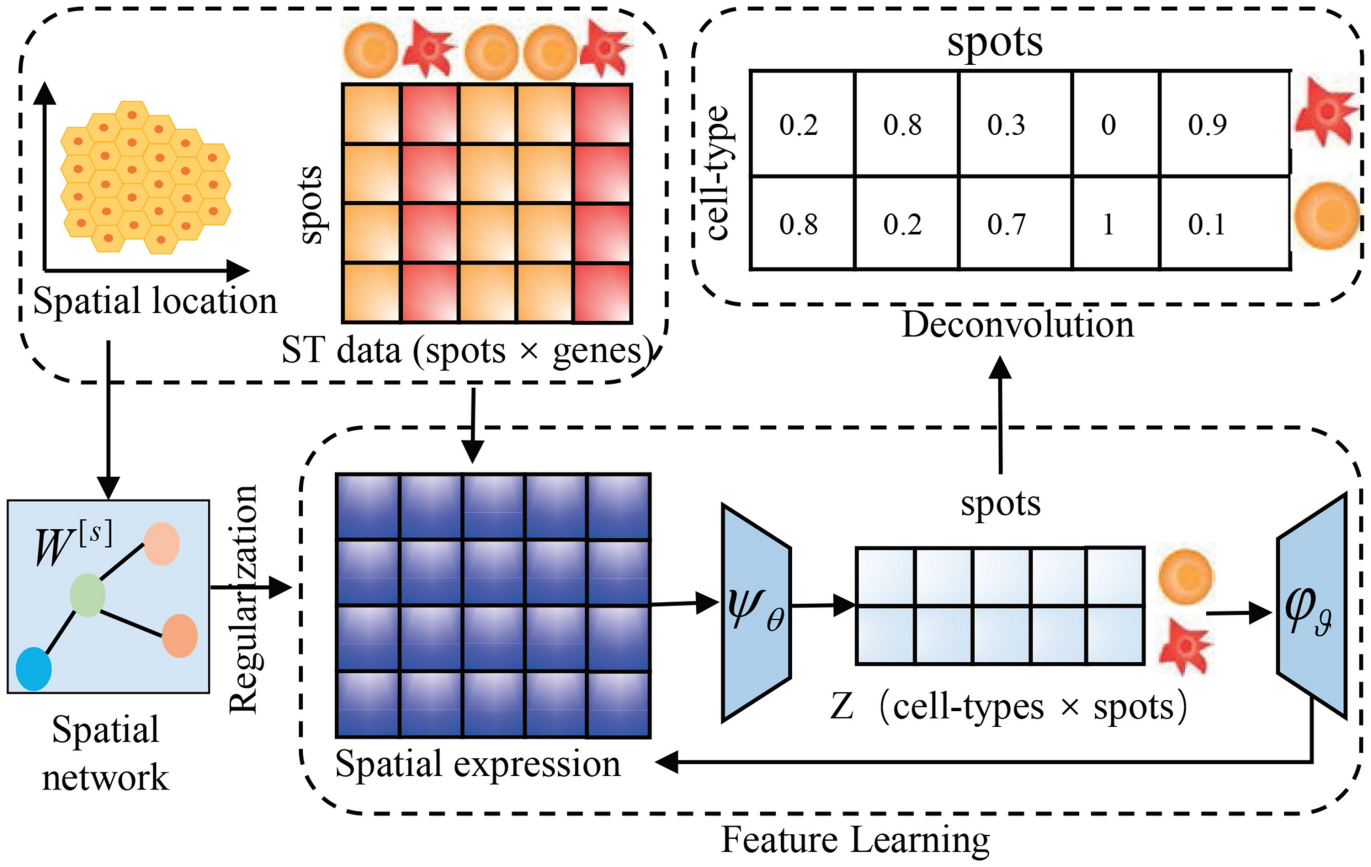
Overview of SA2E. It consists of three major components, i.e. feature learning, construction of signatures of cell types, and cell type deconvolution, where the first procedure learns the low-dimensional features of spots with an auto-encoder by preserving local topological structure of spatial graph of spots. The second procedures learns signatures of cell types by approximating the transcriptional profiles of ST data, which is directly derived from decoder. Finally, SA2E performs cell type deconvolution from the learned feature *Z*.

To address this limitation, we develop a novel spatial-aware spatial-aware auto-encoder algorithm (SA2E) for cell type deconvolution of ST data, where scRNA-seq data and biomarkers of cell types are not required. As shown in Fig. 1, SA2E consists three major components, i.e. feature learning, construction of signatures of cell types and cell type convolution. Specifically, SA2E adopts an auto-encoder to decompose transcriptional profiles of spots of ST data, where spatial information is integrated via graph regularization. In this case, features of spots comprise transcriptional and spatial information. Then, SA2E learns signatures of cell types in terms of spots rather than genes that are widely used by current algorithms, which is fulfilled by enforcing the learned signatures of cell types approximately transcriptional profiles of ST data. Finally, Experimental results demonstrate that SA2E outperforms state-of-the-art baselines for cell type deconvolution, proving that spots can also serve as biomarkers for signatures of cell types.

In summary, the main contributions of this study are listed as

- First, SA2E provides a novel strategy to construct spot-wise cell-type signatures, which does not rely on manually curated cell-type biomarkers and uses scRNA-seq only for simulation-based supervision, thereby extending the applicability of the proposed algorithm for cell-type deconvolution.
- Second, SA2E is a flexible framework for jointly learning signatures of cell types and estimating abundance of cell types for each spot of ST data, which is formulated as an optimization problem.
- Third, SA2E outperforms current baselines on the simulated and real ST datasets generated from different species, tissues and platforms, demonstrating that spots are also promising for serving as biomarkers of cell types.

## 2 Materials and methods

### 2.1 Data pre-processing

For the sc/snRNA-seq datasets, the top 5000 highly variable genes (HVGs) using Seurat v3 (Stuart *et al.* 2019) are selected. Then, the library-size normalization, logarithmic transformation, and scaling are performed with Scanpy (Wolf *et al.* 2018). For the ST datasets, we use Scanpy for pre-processing to obtain normalized spot-level expression profiles. The scRNA-seq dataset is adopted for the generation of simulated ST dataset to testifying performance of SA2E, whereas real ST datasets to validate performance of algorithms for cell type deconvolution. For the ST datasets, we use Scanpy for pre-processing to obtain normalized spot-level expression profiles. The scRNA-seq dataset is adopted to generate a simulated ST dataset for testing the performance of SA2E. In addition, real ST datasets are used to validate the performance of different algorithms for cell-type deconvolution. Notably, the scRNA-seq reference and the corresponding real ST data are derived from the same tissue/source, which helps ensure that the simulated spot compositions are compatible with the underlying spatial structure.

### 2.2 Datasets

Four ST datasets, including one simulated and three real ST data, are selected to validate performance of various algorithms. The simulated dataset is generated from a mouse olfactory bulb (MOB) scRNA-seq reference by aggregating single cells with known labels into spatial spots under a prescribed mixing scheme, providing ground-truth spot-wise compositions for quantitative assessment. The three real ST datasets, including the Legacy ST dataset of mouse olfactory bulb, human breast cancer section and a mouse brain section, are from publicly available studies (Wu and Swarbrick 2021, Hajdarovic *et al.* 2022, Li *et al.* 2022).

### 2.3 Mathematical model of SA2E

#### 2.3.1 Spatial network construction

Each ST dataset can be represented by a tuple (X,Y) of $n_{g}$ genes and $n_{s}$ spots, where $X\in R^{n_{s}\times n_{g}}$ is the expression profile of spots and $Y\in R^{n_{s}\times 2}$ denotes spatial location of spots in terms of 2D coordinates. For each ST dataset, SA2E constructs a spatial graph of spots, denoted by G=(V,E), using K-nearest neighborhood (KNN) with spatial coordinates, where edges weights correspond to negative exponential function of Eucilidean distance of spots. Notice that the number of neighbors sets as 6 according to our previous study (Zha *et al.* 2025).

#### 2.3.2 Cell type feature learning

SA2E employs an auto-encoder to learn low-dimensional features of spots from the high-dimensional expression profiles of ST datasets. Specifically, given expression profiles of spots *X*, we set the number of cell types *k* according to the cell-type annotations obtained from clustering of the corresponding scRNA-seq data. The encoder procedure then obtains features for *k* cell types of spots $Z\in R^{n_{s}\times k}$ as


(1) $$Z=\psi_{\theta }(X),$$

where θ corresponds to parameters of encoder ψ. The decoder reconstructs the original input as


(2) $$\hat{X}=\varphi_{\vartheta }(Z),$$

where ϑ is parameters of decoder φ. The auto-encoder is trained by minimizing the reconstructing error, i.e.


(3) $$∥X-\hat{X}{∥}^{2},$$

where ∥X∥ is the Fronbenious-norm of matrix *X*, i.e. $∥X∥=\sqrt{\sum_{ij}x_{ij}^{2}}$.

We expect that the learned features of spots *Z* also preserves local topological structure of spatial graph *G*, which is fulfilled with Laplacian regularization (He and Niyogi 2003) as


(4) $$Tr(Z^{{\;}^{\prime }}L_{G}Z),$$

where $Tr(X)=\sum_{i}x_{ii}$ is the trace of matrix *X*, $Z^{{\;}^{\prime }}$ is the transpose of matrix *Z*, and $L_{G}=D-W$ is the Laplacian matrix of graph *G* (*W* is the adjacent matrix, and *D* is the degree diagonal matrix), respectively.

A central part of cell-type deconvolution is learning cell-type signatures that link spot-level gene expression to underlying cell types. While scRNA-seq provides rich gene-expression signatures, they may not transfer cleanly to ST because each spot mixes multiple cells and reflects local tissue context. SA2E addresses this by using simulated ST data with known compositions for label-guided training and then adapting the same model to real ST data with reconstruction and a spatial graph regularizer.

Let $X_{s}\in R^{n_{s}\times n_{g}}$ denote the simulated ST expression matrix. The simulated data provide spot-wise cell-type proportions $P\in R^{n_{s}\times k}$, where *k* is the number of cell types and each row of *P* gives the composition of a spot. In addition, the first reconstruction term is formulated with the $L_{1}$ norm to better preserve the sparsity of spatial transcriptomics data. According to Equations (3 and 4), the objective is


(5) $$\underset{\theta ,\vartheta }{\min}\;∥X_{s}-\psi_{\theta }(\varphi_{\vartheta }(X_{s})){∥}_{1}+∥P-Z{∥}_{1}+\alpha \cdot Tr(Z^{{\;}^{\prime }}L_{G^{\left[s\right]}}Z)$$

where $L_{G^{\left[s\right]}}$ is the Laplacian matrix of graph $G^{\left[s\right]}$, $G^{\left[s\right]}$ is the spatial network of simulated ST data according to Spatial network construction. For the real ST expression matrix $X_{r}\in R^{n_{s}\times n_{g}}$, spot-wise proportions are unavailable. We therefore remove the supervision term, and the objective is


(6) $$\underset{\theta ,\vartheta }{\min}\;∥X_{s}-\psi_{\theta }(\varphi_{\vartheta }(X_{r})){∥}_{1}+\alpha \cdot Tr(Z^{{\;}^{\prime }}L_{G^{\left[r\right]}}Z)$$

where $L_{G^{\left[r\right]}}$ is the Laplacian matrix of graph $G^{\left[r\right]}$, $G^{\left[r\right]}$ is the spatial network of real ST data according to Spatial network construction.

Notably, after these two training stages, the latent code *Z* serves as a spot-by-cell-type signature. To obtain the final deconvolution output, we apply a row-wise normalization to *Z* so that each row sums to one. Then the resulting matrix gives the estimated cell-type proportions for each spot. In parallel, the decoder weights define a complementary cell-type-by-gene signature.

#### 2.3.3 Implementation and optimization

SA2E is implemented in Python using the PyTorch framework. All learnable parameters are optimized with the Adam optimizer (Kingma and Ba 2015) with a learning rate of $1\times 10^{-4}$.

### 2.4 Performance evaluation

For the simulated dataset, the truth ground abundance of cell types of each spot is known, four measurements, such as Pearson correlation coefficient (PCC), structural similarity index (SSIM), Jensen–Shannon (JS) divergence and root mean squared error (RMSE), are selected to quantify similarity between the predicted and truth abundance of cell types. Specifically, PCC quantifies the linear agreement across spots (higher is better) and SSIM assesses how well the spatial structure of cell type mapping is preserved (higher is better). JS divergence measures discrepancies between the predicted and reference composition of cell types (lower is better), whereas RMSE models distance between them (lower is better). For the real ST datasets, we set the anatomical or pathological annotations as ground truth and adopt adjusted Rand index (ARI) and cluster purity (higher is better).

## 3 Experiments and results

### 3.1 Overview of SA2E for cell type deconvolution

Deciphering abundance of cell types for spots of ST data is promising since it improves resolutions of spots. Current algorithms explicitly hypothesize that biomarkers of cell types are comprehensive and available. However, these algorithms fail to execute if bio-makers of cell types are unavailable or incomprehensive, hampering the application of current approaches. Unlike available algorithms, we propose a novel spatial-aware auto-encoder algorithm (SA2E) for cell type deconvolution without requiring biomarkers of cell types.

Actually, SA2E simultaneously infers signatures of cell types and estimates mixtures of cell types, whereas available state-of-the-art baselines only focus on the latter task. To address the first concerning, SA2E learns signatures of cell types from features of spots, where biomarkers of cell types are automatically selected, thereby enhancing quality of signatures of cell types. To attack the second concerning, SA2E employs an auto-encoder to learn features of spots by integrating spatial and transcriptional information of spots with the learned signatures of cell types. In this case, SA2E successfully overcomes limitations of the absence of biomarkers and scRNA-seq data by fully exploiting knowledge of ST data.

Figure 1 presents an overview of SA2E, which consists of three major components, i.e. feature learning, constructing signatures of cell types, and cell type deconvolution. SA2E takes an auto-encoder to learn the low-dimensional features of spots from transcriptional profiles of spots by minimizing the reconstructing error (Section 2). To fully integrate spatial information of spots, SA2E constructs spatial graph of spots with KNN in terms of Euclidean distance of spatial coordinates of spots, where the learned features preserve local topological structure of the spatial graph with regularization. In this case, SA2E improves quality of spot features because spatial and transcriptional information is implicitly fused under the auto-encoder framework. Then, SA2E learns signatures of cell types, which can be directly derived from decoder by enforcing signatures of cell types to reconstruct the original transcriptional profiles. In this case, signatures of cell types are highly associated with features of spots, thereby successfully avoiding the limitations of biomarkers. Finally, SA2E is formulated as a joint optimization problem, providing a more flexible and applicable framework for cell type deconvolution of ST data without requiring biomarkers of cell types.

### 3.2 Benchmarking SA2E with simulated ST dataset

We first validate performance of SA2E with the simulated dataset, where the layout and mixtures of spots are known. The simulated dataset is widely adopted as benchmarking (Ma and Zhou 2022, Zha *et al.* 2025), which is derived from a mouse olfactory bulb (MOB) scRNA-seq dataset and are aggregated into spatial spots with the prescribed mixing scheme (Ma and Zhou 2022). Because the ground-truth composition of spots is known, we select four measurements, including PCC, SSIM, JS divergence and RMSE, to fully validate performance of various algorithms (Section 2). Nine representative algorithms, such as SPOTlight (Elosua-Bayes *et al.* 2021), RCTD (Cable *et al.* 2022), SpatialDWLS (Dong and Yuan 2021), Cell2location (Kleshchevnikov *et al.* 2022), CARD (Ma and Zhou 2022), Redeconve (Zhou *et al.* 2023), DestVI (Lopez *et al.* 2022), DSTG (Song and Su 2021), and OmicsTweezer (Yang *et al.* 2025) are selected as baselines due to their excellent performance, where the first four algorithms belong to independent-based category, and others to integration-based one. Other algorithms are excluded because our previous study (Zha *et al.* 2025) demonstrate that they are inferior to these selected ones.

**Figure 2:**
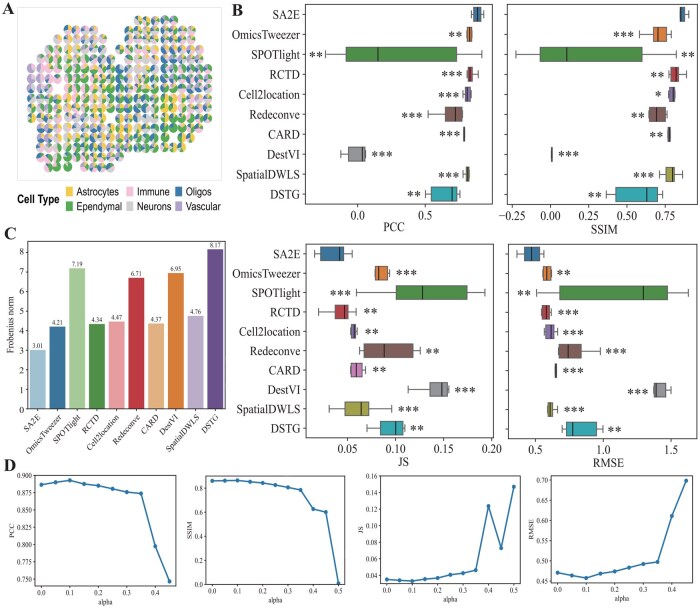
Performance of various algorithms for cell type deconvolution on the simulated dataset. (A) Visualization of the simulated dataset, where each spot is depicted as a pie chart to illustrate distribution of cell types, (B) performance of various algorithms for cell type deconvolution on the simulated dataset in terms of various measurements, including PCC (left-top panel), SSIM (top-right panel), JS divergence (bottom-left panel), and RMSE (bottom-right panel), respectively. Significance is obtained with one-sided Student’s *t*-test and corrected by Benjamini–Hochberg (FDR-BH) test. */**/*** denote *P*-value <5.0E-1/5.0E-2/5.0E-3, respectively, (C) distance between the predicted and ground truth abundance of cell types in terms of Frobenius norm, and (D) how parameter α effects performance of SA2E in terms of various measurements.

Figure 2A visualizes distribution of cell types of spots in simulated dataset, where each spot corresponds to a pie chart to depict abundance of cell types. We validate performance of all these algorithms by estimating abundance of cell types for each spot in the simulated dataset. To fully validate performance of algorithms, four typical measurements, including PCC, SSIM, JS divergence, and RMSE, are selected (Section 2). Figure 2B depicts performance of various algorithms for cell types deconvolution on the simulated dataset in terms of these four measurements, where top-left pane is for PCC, top-right for SSIM, bottom-left for JS divergence, and bottom-right for RMSE, respectively. From these panels, it is easy to conclude that SA2E achieves the best performance on the simulated dataset in terms of all these measurements. For example, PCC of SA2E is 0.89±0.04 (mean ± standard deviation), whereas it is 0.83±0.05 (OmicsTweezer), 0.28±0.52 (SPOTlight), 0.84±0.03 (RCTD), 0.83±0.05 (Cell2location), 0.69±0.10 (Redeconve), 0.80±0.04 (CARD), 0.82±0.03 (SpatialDWLS), and 0.52±0.41 (DSTG), respectively.

Moreover, it is observed that SA2E often achieves the best performance, followed by RCTD and Cell2location. SPOTlight and DestVI obtain the worst performance. The reason why SPOTlight and DestVI cannot precisely estimate abundance of cell types of spots because the adopted linear model is sensitive to distributions of cell types, i.e. they achieve an excellent performance if and only if all cell types are equally distributed. RCTD and Cell2location are much better than SPOTlight because these algorithms employ additional information to infer the abundance of cell types. Furthermore, the standard deviation of SA2E is 0.04, which is much less than others, showing that the proposed algorithm is also robust. There are three reasons explain why SA2E outperforms all these baselines. First, signatures of cell types is learned, where biomarkers of cell types are also automatically selected, thereby improving quality of signatures of cell types. In this case, the most discriminative biomarkers of cell types can be identified to facilitate the estimation of cell types of each spot. Second, SA2E jointly learns features of spots and signatures of cell types, where features of objects is incorporated into information of signatures of cell types, thereby enhancing quality of features to discriminate cell types of spots. Third, the proposed algorithm enforces the learned signatures of cell types to approximate the transcriptional profiles of ST data, further deepening connection between signatures and features of spots. To further quantify performance of algorithms, we also take Fronbenius norm of difference of the predicted and truth abundance of spot-cell type matrix as shown in Fig. 2C. SA2E obtains the minimal Fronbenius norm, demonstrating that the abundance of cell types predicted is very close to the truth ground one.

Finally, we also investigate parameter effect of SA2E, which involves one parameter α to control importance of local structure preservation. Figure 2D depicts how performance of SA2E changes in terms of all these four measurements as parameter α increases. We conclude that SA2E achieves the best performance if parameter α≤0.3, and then performance of SA2E begins to decrease as parameter α. The reason is that when parameter α is too large, spatial information of features dominates transcriptional information, thereby reducing quality of features of spots. SA2E reaches a good trade-off between spatial and transcriptional information at parameter α= 0.1. These results demonstrate that learning signatures of cell types is promising for cell type deconvolution of simulated dataset.

### 3.3 Benchmarking various algorithms with mouse brain ST dataset

Previous experiment validate performance SA2E on the simulated, and we further testify its performance for cell type deconvolution with biological ST dataset. Specifically, the mouse brain ST dataset with neural cell types organized in spatial architecture, is selected, where 59 manually annotated cell types are available (Hajdarovic *et al.* 2022). By following the previous studies (Elosua-Bayes *et al.* 2021, Kleshchevnikov *et al.* 2022, Zha *et al.* 2025), we only select the most dominant 9 cell types for deconvolution, including Oligo_2, Ext_Thal_1, Ext_L25, Inh_Meis2_3, Ext_L56, Ext_L5_2, Inh_4, Ext_L6B, and Ext_L23.

**Figure 3:**
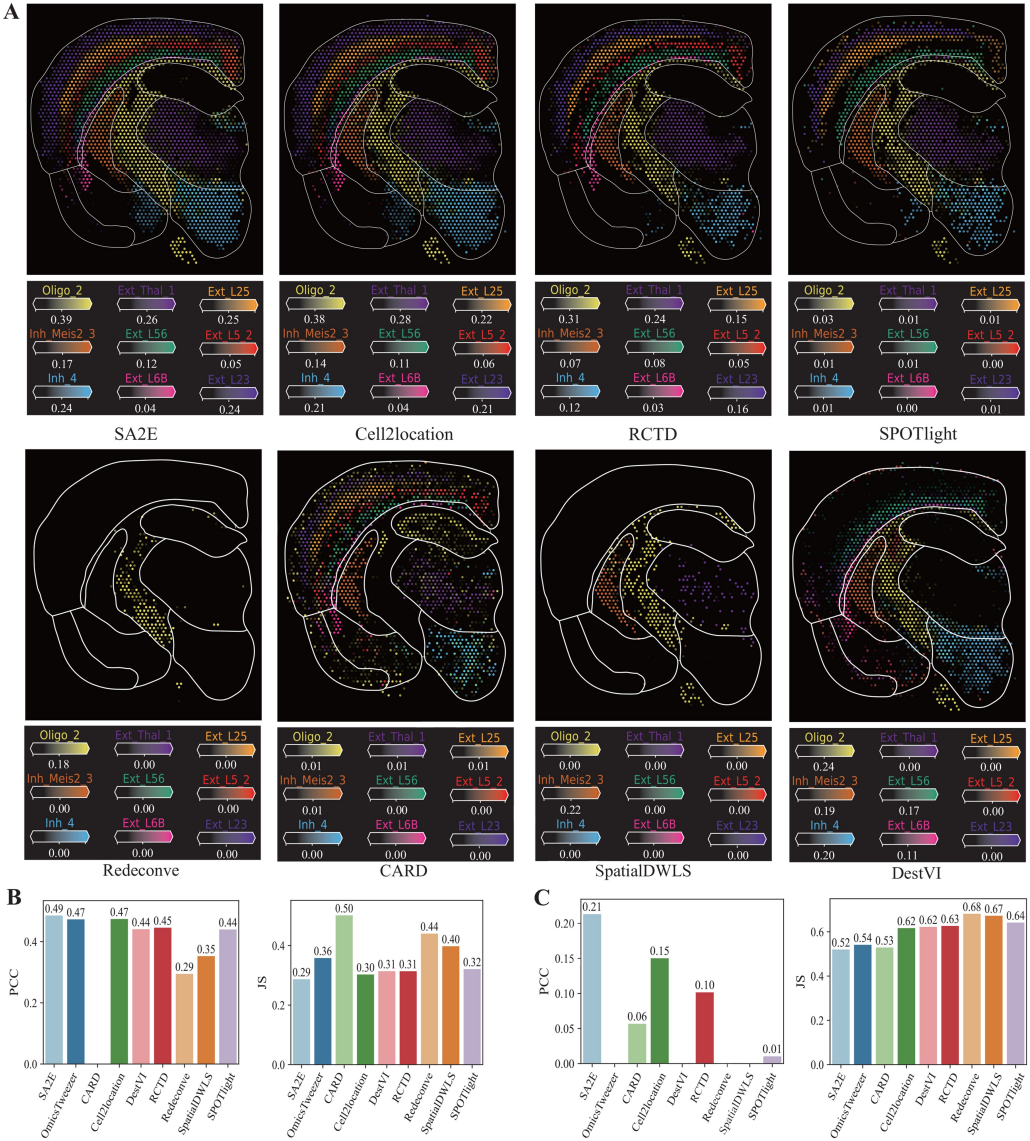
Performance of algorithms for cell type deconvolution on the mouse brain ST dataset. (A) Visualization of results of region-specific cell types obtained by various algorithms in the mouse brain ST dataset, where regions are enclosed by white lines, (B) PCC and JS of abundance of cell type Oligo_2 with marker gene *Prr5l* obtained by different algorithms, and (C) PCC and JS of abundance of cell type Ext_L5_2 with marker gene *Gm28928*, respectively. For panels (B) and (C), any PCC values reported as zero arise from truncating negative values to zero for display.

Since mixtures of cell types of spots are unknown, we cannot directly quantify performance of various algorithms for cell type deconvolution of mouse brain ST data. We first check whether cell type deconvolution of spots facilitate the identification of specific regions. Figure 3A visualizes specific regions identified by various algorithms for the mouse brain ST data, where regions are surrounded by white lines based on results of cell type deconvolution predicted by different algorithms. It is easy to observe that SA2E and Cell2location precisely recognize these nine cell types, whereas other baselines fail to identify all these nine cell types. For example, CARD only identifies cell type Oligo_2 and fails to identify other eight cell types. These panels demonstrate that SA2E can also be applied to mouse brain ST data for cell type deconvolution, which facilitates the identification of specific regions of ST data.

To quantify performance of various for cell type deconvolution on the mouse brain dataset, we manually annotation of spots of the oligodendrocyte subtype Oligo_2 and its marker gene *Prr5l* as our previous study (Zha *et al.* 2025). Figure 3B describes performance of various algorithms for cell type deconvolution of the oligodendrocyte subtype Oligo_2, where left panel is for PCC and right for JS. It is easy to observe that SA2E achieves the best performance. For instance, PCC of SA2E is 0.49, whereas it 0.47, 0.47, 0.44, 0.45, 0.29, 0.35, 0.44 for OmicsTweezer, Cell2location, DestVI, RCTD, Redevonve, SpatialDWLS, and SPOTlight, respectively. These results demonstrate that SA2E also precisely estimates abundance of cell types of spots in mouse brain ST dataset. To check whether SA2E is sensitive to the oligodendrocyte subtype Oligo_2. Analogously, we perform analysis on the cell type Ext_L5_2 with marker gene *Gm28928*. As shown in Fig. 3C, where SA2E also achieves the best performance.

These results demonstrate that learning signatures of cell types is also applicable for the mouse brain dataset.

### 3.4 Benchmarking various algorithms with breast cancer ST dataset

**Figure 4:**
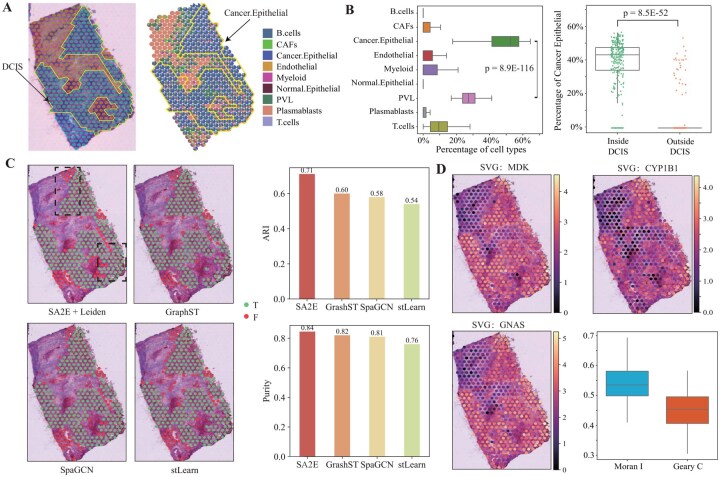
Performance of various algorithms for cell type deconvolution on the human breast cancer ST dataset. (A) Visualization of H&E image of the breast cancer with the DCIS region surrounded yellow solid line (left), and results of cell type deconvolution obtained by SA2E (right), (B) Distributions of cell types of spots within DCIS region (left), and distribution of percentages of cancer epithelial cell type inside and outside of DCIS region (right, significance is with the one-sided Student’s *t*-test), (C) Performance of various algorithms on the identification of the DCIS domain based on the results of cell type deconvolution (left), and ARI and purity of various algorithms of spots within DCIS region (right), and (D) visualization of genes within spatial domain (MDK, CYP1B1, and GNAS) and Moran’s I and Geary’s C statistic analysis, respectively.

Previous experiment demonstrates that SA2E performs cell type deconvolution on ST data of normal tissues, and then we further validate performance of SA2E with cancer ST dataset. Specifically, we selected a breast cancer ST dataset generated using the 10× Genomics Visium platform (Wu *et al.* 2021), which contains a ductal carcinoma in situ (DCIS) region. Figure 4A left panel visualizes the H&E image with the DCIS region surrounded by solid yellow lines.

SA2E is applied to the breast cancer dataset and the result of cell type deconvolution is visualized in Fig. 4A right panel, where each spot is represented by a pie chart. The comparison of these two panels of Fig. 4A demonstrates that SA2E precisely identifies DCIS, showing that the predicted abundance of cell types of spots accurately preserves structure of cancer region DCIS. Since DCIS region is highly related to breast cancer, we compare all these cell types of spots, including B cells, CAFs, Cancer Epithelial, Endothelial, Myeloid, Normal Epithelial, PVL, Plasmablasts and T cells, within DCIS predicted by SA2E, where distributions of them are shown in Fig. 4B left panel. It is easy to observe that the cancer epithelial cell type dominates others (*P*-value = 8.9E-116, one side Student’s *t*-test), demonstrating cell type deconvolution precisely identifies cancer-related cell types. We also compare percentages of cancer epithelial cell type inside and outside of DCIS (Fig. 4B right panel, *P*-value = 8.5E-52, one side Student’s *t*-test), whereas spots outside of DCIS have much less cancer epithelial cell type.

Then, we further examine whether deconvolution of cell types through SA2E enhance performance of the identification of DCIS, where Leiden algorithms is used for clustering. Figure 4C left panels visualize the DCIS region identified by SA2E, GraphST, SpaGCN, and stLearn, where green indicates the correctly identified spots, otherwise red. Notably, SA2E is more accurate than baselines, where is measured by ARI and purity as shown in Fig. 4C right panel. ARI and Purity of SA2E are 0.71 and 0.84, which are much higher than those of baselines, proving that SA2E is more precise on the estimation of cell types of spots. Furthermore, we also visualize spatial distributions of bio-marker gene MDK, CYP1B1, and GNAS, which are coherent with respect to DCIS region (Fig. 4D left panel), and the Moran’s I and Geary’s C statics further prove that these SVGs are highly related to breast cancer (Fig. 4D right panel).

### 3.5 SA2E is applicable for spatial transcriptomics datasets generated by various platforms

**Figure 5:**
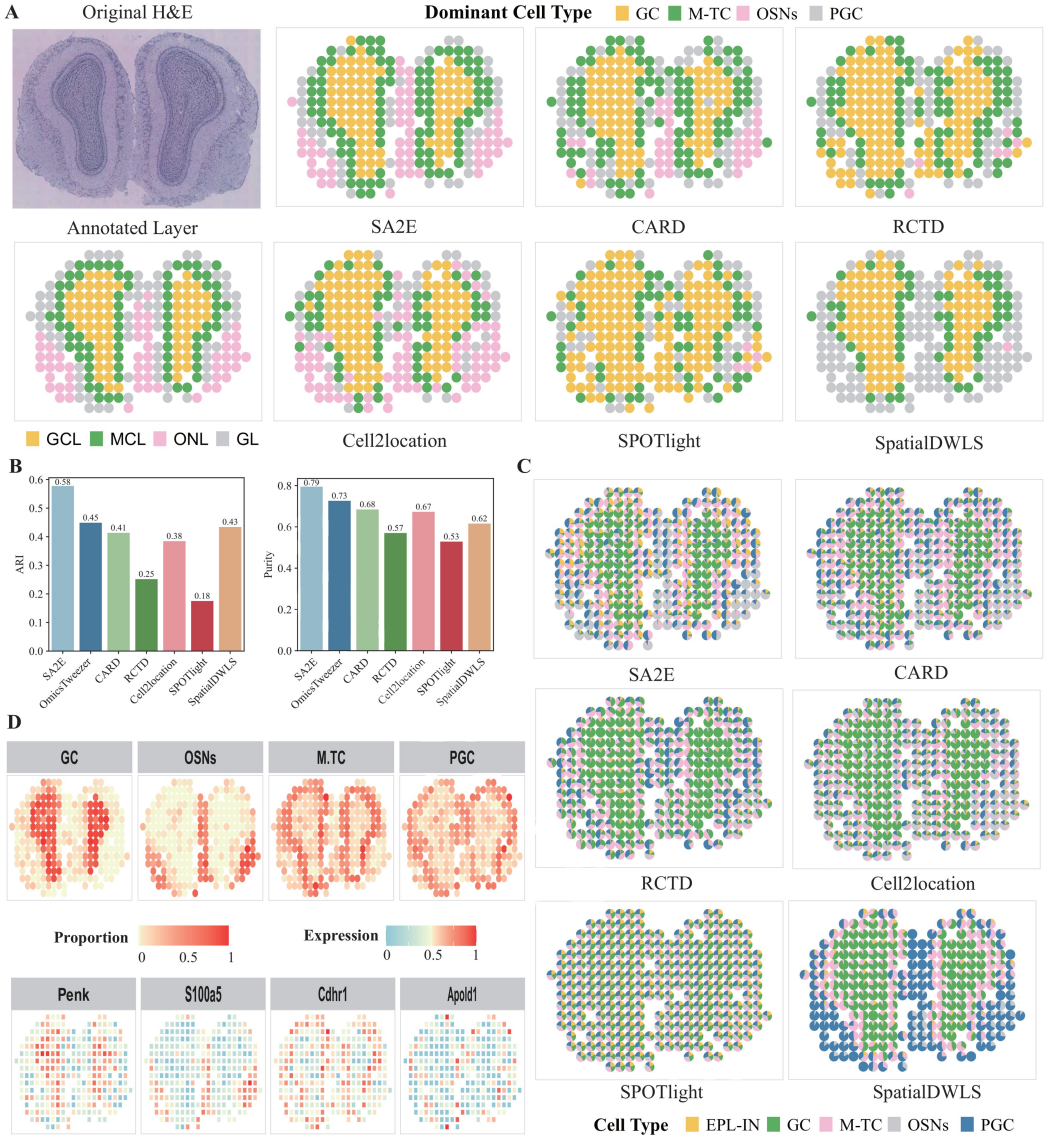
Performance of SA2E on the mouse olfactory bulb (MOB) dataset generated with Legacy platform. (A) Visualization of H&E image and the four anatomical annotated layers, including FCL, MCL, GL, and ONL from inside to outside (left). Performance of algorithms on the identification of spatial domains (right), (B) ARI and purity of algorithms on the MOB dataset, (C) visualization of results of cell type deconvolution obtained by various algorithms, and (D) visualization of spatial distribution of bio-marker genes, respectively.

All these datasets in the previous experiments are generated with 10× Genomics Visium platform, and we select an additional mouse olfactory bulb (MOB) dataset generated on the Legacy platform to testify applicability of SA2E. As shown in Fig. 5A left panel, the MOB dataset is with four major laminae, i.e. granule cell layer (GCL), mitral cell layer (MCL), outer nerve layer (ONL), and glomerular layer (GL).

Visualization of spatial domains identified by various algorithms is shown in Fig. 5B, where SA2E is much better than baselines because spatial domains identified by SA2E are consistent with the annotation of granule cells (GC), mitral/tufted cells (M-TC), olfactory sensory neurons (OSNs), and periglomerular cells (PGC). In contrast, these algorithms cannot discriminate domains across layer boundaries or fragmented patches within individual layers. For example, CARD and RCTD mix GC and M-TC in the inner bulb, whereas Cell2location, SPOTlight and SpatialDWLS fail to discriminate OSN and PGC. These results show that SA2E is also applicable to Legacy platform.

To quantify the agreement with these annotated layers and identified ones, ARI and cluster purity of various algorithms is shown Fig. 5B, where SA2E achieves the best performance. Specifically, ARI and purity of SA2E 0.58 and 0.79, which are much higher than those of baselines. Then, we further visualize results of cell type deconvolution obtained by various algorithms as shown in Fig. 5C, where pie charts illustrate abundance of cell types of spots. The results obtained SA2E show smooth, layer-specific gradients, GC fractions gradually decrease from the inner to outer layers, M-TC concentrate near the mitral band, and OSNs/PGC progressively increase toward the bulb surface, matching the expected layer boundaries. However, these baselines fail to identify these layers, proving that the proposed algorithm precisely estimates abundance of cell types of ST of Legacy platform.

Finally, we examine spatial distribution of bio-marker genes of different cell types, including *Penk* for GC, *S100a5* for OSNs, *Cdh1* for M-TC and *Apold1* for PGC, as shown Fig. 5D, which are highly consistent with structure of cell types. Together, these results suggest that SA2E is insensitive to platforms for ST datasets.

## 4 Conclusion

To address the limitation that many existing cell type deconvolution algorithms require cell-type biomarkers, we propose a novel method, SA2E, which does not require any predefined biomarkers of cell types. SA2E addresses this limitation by automatically learning cell-type signatures from ST data, where the biomarkers of cell types are implicitly captured during signature learning. In this way, SA2E links the learned signatures with ST data, which enhances the quality of spot features and the inferred cell-type biomarkers. Extensive experiments demonstrate that SA2E significantly outperforms baseline methods on both simulated and biological ST datasets. Furthermore, SA2E precisely estimates cell-type abundances across different tissues and species, and it is applicable to ST data generated from various platforms.

In summary, SA2E provides an effective strategy for cell type deconvolution by jointly learning spot features and cell-type signatures, thereby improving deconvolution performance without requiring predefined biomarkers and extending the applicability of the proposed algorithm. We also acknowledge several limitations and promising future directions. First, SA2E is reconstruction driven, and its performance may degrade when ST expression profiles are heavily corrupted by noise, which can compromise faithful reconstruction and subsequent deconvolution. Second, the current framework incorporates only spatial location information. Integrating additional modalities such as histology or imaging features is a natural extension to further improve robustness and accuracy. Finally, accurately estimating rare cell types remains challenging for deconvolution, and addressing this issue is critical for revealing fine-grained biological mechanisms.

## Supplementary Material

btag133_Supplementary_Data

## Data Availability

All datasets used in various experiments can be accessed publicly. Simulated ST data is at https://drive.google.com/drive/folders/1fgv9JIecb_XoQ_FEP77U4zRakrKX9mSn. ST data and snRNA-seq data for mouse brain are at https://www.ebi.ac.uk/biostudies/arrayexpress/studies/E-MTAB-11114. ST data and snRNA-seq data for human breast cancer are at https://zenodo.org/records/4739739 and https://singlecell.broadinstitute.org/single_cell/study/SCP1039/a-single-cell-and-spatially-resolved-atlas-of-human-breast-cancers. Mouse olfactory bulb data is at http://www.spatialtranscriptomicsresearch.org/.
