# Supplementary material for: SA2E: spatial-aware auto-encoder for cell type deconvolution of spatial transcriptomics data
Source: Bioinformatics. 2026 Mar 20;42(4):btag133. doi: 10.1093/bioinformatics/btag133 (PMC13070677; doi:10.1093/bioinformatics/btag133)
Supplement: btag133_Supplementary_Data [file btag133_supplementary_data.pdf]

Supplementary Material for "SA2E: spatial-aware auto-encoder for cell type deconvolution of spatial transcriptomics data"

Yaxiong Ma, Zengfa Dou, Yuhong Zha, Xiaoke Ma

February 28, 2026

Abstract

Supplementary Material provides detailed implementation of the model, extended experiments on additional public datasets (human heart, mouse embryo, and MERFISH), robustness analyses of SA2E, and correlation analyses between the learned signatures and the corresponding reference patterns.

S1: Implementation Details

Auto-encoder architecture and hyperparameters

The encoder is a multilayer perceptron with layer widths `inputdim` $\rightarrow 512 \rightarrow 256 \rightarrow 128 \rightarrow 64 \rightarrow k$, using `CELU` activations, `Dropout` between hidden blocks, and a final `ReLU` to enforce non-negativity of the output. The decoder is a linear stack with layer widths $k \rightarrow 64 \rightarrow 128 \rightarrow 256 \rightarrow 512 \rightarrow$ `inputdim` (with `bias=False`) to reconstruct the input expression profiles. In addition, the SA2E code has been publicly released on GitHub[1], and all implementation details are available in the repository to ensure reproducibility.

Mixing scheme and simulation parameters

The simulated ST dataset is generated from mouse olfactory bulb scRNA-seq by constructing pseudo spots through a reproducible mixing-and-aggregation procedure implemented in our code (see `method/deconvolution.py`). Concretely, we first select the top 5000 highly variable genes (Seurat v3 flavor). Spot-wise cell-type fractions are then sampled from a Dirichlet distribution, either without prior (`d_prior=None`) using $\mathrm{Dir}(\mathbf{1})$, or with a specified prior vector `d_prior`. To mimic compositional sparsity and rare components, the simulator further supports (a) `sparse` spot compositions by randomly zeroing out a subset of cell types for a subset of spots controlled by `sparse_prob`, and (b) `rare` cell-type

[1]`https://github.com/xkmaxidian/SA2E/tree/master`

settings by forcing a subset of cell types to take small proportions (uniformly sampled in a low range) controlled by `rare_percentage`.

To ensure spatial consistency between spot compositions and neighborhood structure, we optionally apply an explicit spatial smoothing step when coordinates are provided (`coords`): a kNN graph is constructed (`knn_k`) and neighbor weights are computed using a Gaussian kernel (`bandwidth`), followed by blending the original and neighbor-averaged compositions with strength `spatial_strength`. The resulting normalized weights also define the simulated spatial graph W returned by the simulator.

Given the finalized proportions, the number of cells per spot is determined either by (a) a fixed total cell count with floor operation (`n` and `rangePick=False`), or (b) random total cells per spot in a specified range (`rangePick=True` with `min_cells_per_spot` and `max_cells_per_spot`), where cell-type counts are drawn by multinomial sampling. Finally, for each spot and each cell type, the simulator samples the corresponding number of single cells with replacement and aggregates their expression to form the spot-level count profile. Randomness is controlled by `random_state`. These details and parameter settings are fully reproducible from the released code.

S2: Extended Experiments on Additional Public Datasets

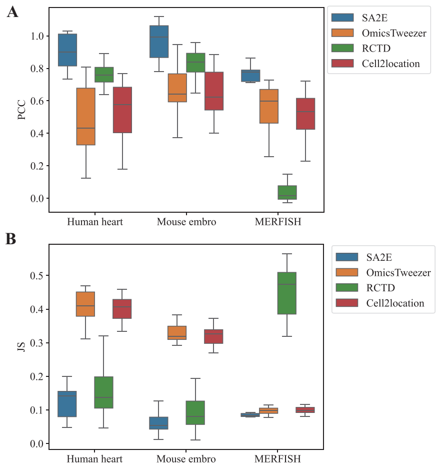

Figure 1: Extended benchmarking on additional public datasets. Performance comparison of SA2E, OmicsTweezer, RCTD, and Cell2location on three datasets, including human heart, mouse embryo, and MERFISH. (A) Boxplots of PCC between estimated and reference cell-type proportions. (B) Boxplots of JS divergence between estimated and reference cell-type proportions.

To further assess generalizability across tissues, species, and platforms, we extended benchmarking to three additional public datasets, including human heart, mouse embryo, and MERFISH, and compared SA2E with OmicsTweezer, RCTD, and Cell2location. As shown in Fig. 1, SA2E yields higher PCC (panel A) and lower JS divergence (panel B) across these datasets, indicating improved agreement with the references under both metrics.

S3: Robustness Analyses

In the main text, we analyzed the sensitivity of SA2E to the spatial regularization weight α. Here, we further examine robustness on the simulated ST dataset with respect to the quality of simulated data (approximated by the cell number per spot), the KNN parameter used for spatial graph construction, and preprocessing choices including spot size and normalization strategy.

S3.1 Sensitivity to simulated ST data quality (cell number per spot). We assess the quality of simulated ST data by varying the cell number per spot, which directly controls the mixing complexity of each pseudo spot. Under different cell-number settings, we evaluate SA2E using JS divergence between the estimated and ground-truth cell-type proportion matrices.

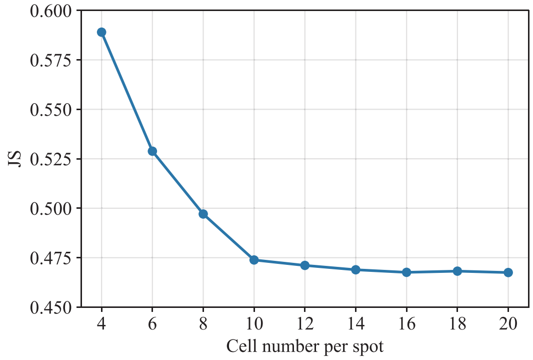

Figure 2: Sensitivity to simulated ST data quality. JS divergence of SA2E on the simulated ST dataset under varying cell number per spot. Lower JS indicates better agreement with the ground truth.

As shown in Fig. 2, JS divergence decreases as the cell number per spot increases, indicating improved agreement between the estimated and ground-truth cell-type proportions. The improvement becomes marginal beyond approximately 10 cells per spot, suggesting that SA2E remains stable once pseudo spots achieve a moderate level of mixing complexity.

S3.2 Sensitivity to the KNN parameter. We investigate the effect of the KNN neighborhood size k (denoted as `knn_k`) used to construct the spatial

neighborhood graph. For each candidate k, we re-run SA2E on the simulated dataset and report performance in terms of PCC, SSIM, JS divergence, and RMSE.

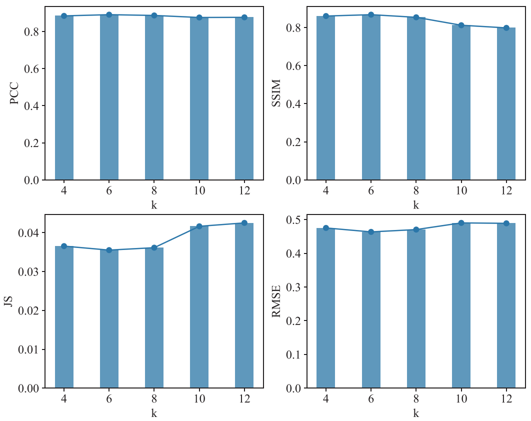

Figure 3: Sensitivity analysis of the KNN parameter for spatial graph construction. Performance of SA2E on the simulated ST dataset under different values of the KNN neighborhood size k. The deconvolution accuracy is evaluated using PCC, SSIM, JS divergence, and RMSE.

As shown in Fig. 3, SA2E exhibits stable performance across a range of k values. PCC and SSIM remain consistently high, while JS divergence and RMSE show only minor variations, indicating that SA2E is not overly sensitive to the choice of k within a reasonable range.

S3.3 Sensitivity to spot size and normalization strategy. We further evaluate the robustness of SA2E to preprocessing choices on the simulated ST dataset. Specifically, we examine (i) spot size and (ii) normalization strategy, including min–max scaling (`mms`), max-abs scaling (`mas`), and standard scaling (`ss`, i.e., z-score).

As shown in Fig. 4**A**, SA2E maintains consistently high PCC and low JS divergence across different spot-size settings, indicating stable performance under varying mixing scales. Fig. 4**B** shows that SA2E achieves comparable performance under `mms` and `mas`, while standard scaling (`ss`) leads to slightly lower PCC and higher JS in our experiments.

S4: Correlation Analysis of Learned Signatures

We perform correlation analyses of the cell-type signatures learned by SA2E on the Human Breast Cancer, MOB, mouse brain, and simulated ST datasets to examine whether different cell types are associated with distinct signature patterns.

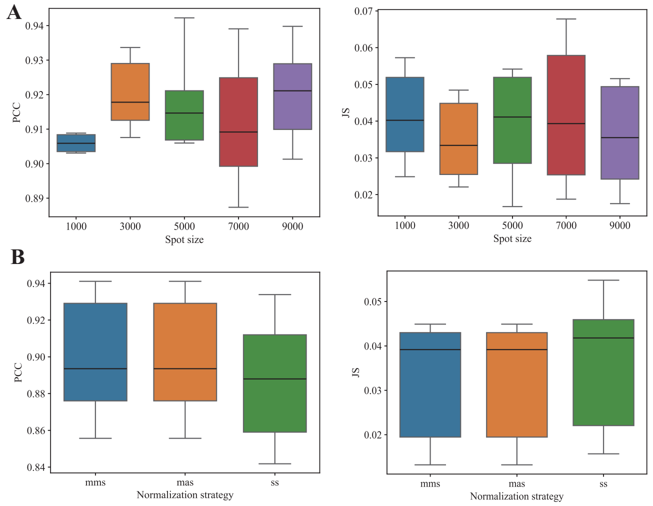

Figure 4: Robustness analysis with respect to spot size and normalization strategy on the simulated ST dataset. **(A)** Sensitivity to spot size. Boxplots report PCC (left) and JS divergence (right) under different spot-size settings. **(B)** Sensitivity to normalization strategy. Boxplots report PCC (left) and JS divergence (right) under three normalization strategies, including min–max scaling (**mms**), max-abs scaling (**mas**), and standard scaling (**ss**, i.e., z-score).

As shown in Fig. 5, the correlation structure is cell-type specific, with relatively higher correlations concentrated within related immune lineages (e.g., B-cells, plasmablasts, and T-cells) and lower correlations observed between immune and epithelial compartments (Cancer Epithelial and Normal Epithelial).

As shown in Fig. 6, higher correlations are concentrated along the diagonal, while most off-diagonal entries are comparatively lower, indicating that SA2E learns distinguishable signatures across MOB cell types.

As shown in Fig. 7, diagonal entries tend to be higher than most off-diagonal entries, suggesting that SA2E learns distinct signatures across the considered mouse brain cell types.

As shown in Fig. 8, higher correlations are mainly concentrated along the diagonal, while most off-diagonal entries are relatively lower, indicating distinguishable signatures across simulated cell types under controlled settings.

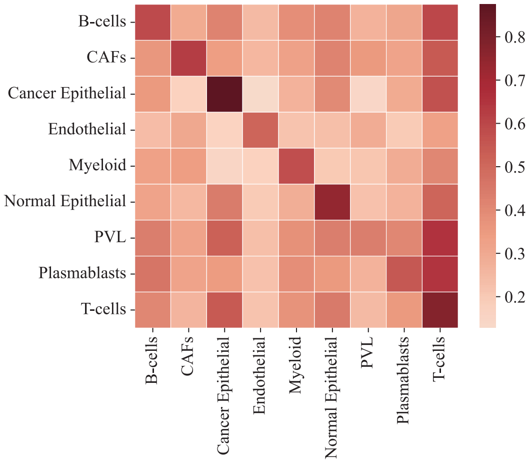

Figure 5: Correlation analysis of learned cell-type signatures on the Human Breast Cancer dataset. Heatmap of pairwise correlations between cell-type signatures learned by SA2E across major cell types (B-cells, CAFs, Cancer Epithelial, Endothelial, Myeloid, Normal Epithelial, PVL, Plasmablasts, and T-cells). Darker colors indicate higher correlation values.

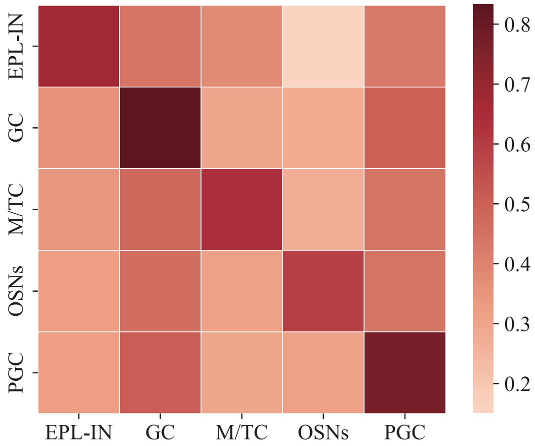

Figure 6: Correlation analysis of learned cell-type signatures on the MOB dataset. Heatmap shows pairwise correlations between the cell-type signatures learned by SA2E for major MOB cell types (EPL-IN, GC, M/TC, OSNs, and PGC). Darker colors indicate higher correlation values.

S5: Ablation Study of Spatial Graph Regularization

In the main text, we report a sensitivity analysis of the spatial graph regularization weight α on the simulated ST dataset, where $\alpha = 0$ corresponds to

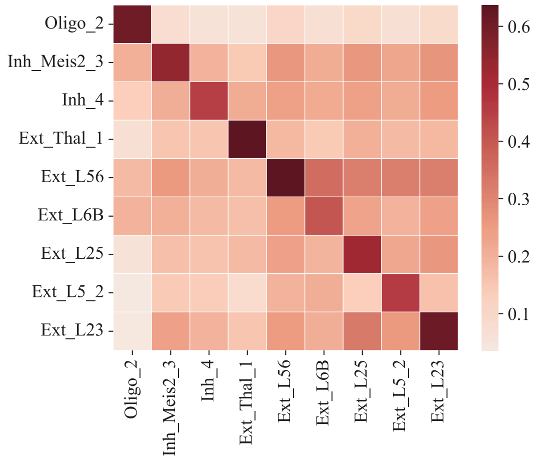

Figure 7: Correlation analysis of learned cell-type signatures on the mouse brain dataset. Heatmap shows pairwise correlations between the cell-type signatures learned by SA2E across major mouse brain cell types (e.g., Oligo_2, inhibitory neuron subtypes, and excitatory neuron subtypes). Darker colors indicate higher correlation values.

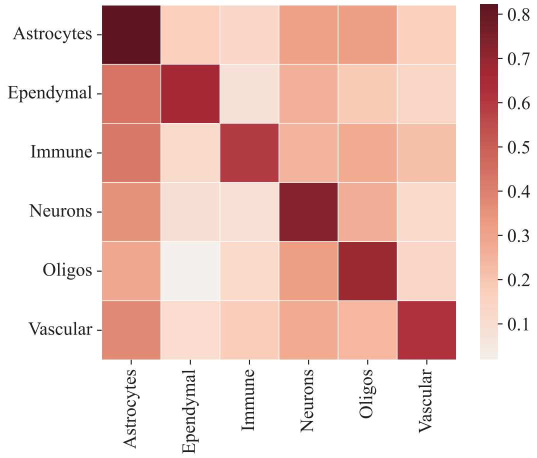

Figure 8: Correlation analysis of learned cell-type signatures on the simulated ST dataset. Heatmap shows pairwise correlations between the cell-type signatures learned by SA2E across major simulated cell types (Astrocytes, Ependymal, Immune, Neurons, Oligos, and Vascular). Darker colors indicate higher correlation values.

removing the spatial graph term. In this section, we provide additional ablation results and analyses to further quantify the contribution of the spatial constraint. Specifically, we report (i) $\alpha = 0$ ablations on three real datasets (mouse

brain, breast cancer, and MOB), (ii) the interaction between α and data characteristics (tissue complexity and spot density) on the simulated dataset, and (iii) the effect of alternative spatial graph construction strategies.

S5.1 Ablation with $\alpha = 0$ on real ST datasets

Mouse brain. We evaluate SA2E with the spatial graph term removed ($\alpha = 0$) and compare it with the default setting ($\alpha = 0.1$) on the mouse brain dataset. As shown in Fig. 9, enabling spatial graph regularization improves PCC and reduces JS divergence for representative cell types.

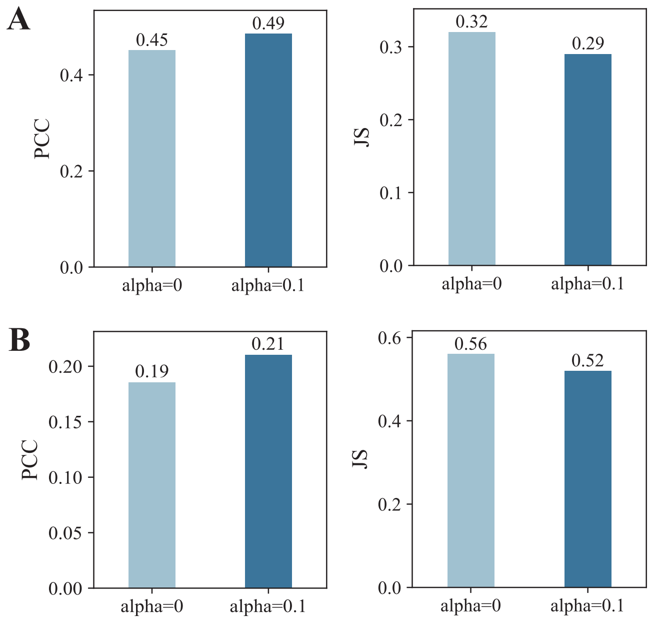

Figure 9: **Ablation on the mouse brain dataset.** Comparison of SA2E without spatial graph regularization ($\alpha = 0$) and the default setting ($\alpha = 0.1$) on two representative cell types. **(A)** Oligo_2 (marker gene *Prr5l*): PCC increases from 0.45 to 0.49 and JS decreases from 0.32 to 0.29 when enabling the spatial graph term. **(B)** Ext_L5_2 (marker gene *Gm28928*): PCC increases from 0.19 to 0.21 and JS decreases from 0.56 to 0.52 when enabling the spatial graph term.

Breast cancer (DCIS identification). We further compare SA2E with $\alpha = 0$ and $\alpha = 0.1$ on the breast cancer dataset using DCIS identification as the downstream evaluation. As shown in Fig. 10, removing the spatial graph regularization reduces both ARI and purity.

MOB (spatial domain identification). On the MOB dataset, we evaluate the impact of removing the spatial graph term using spatial domain identifica-

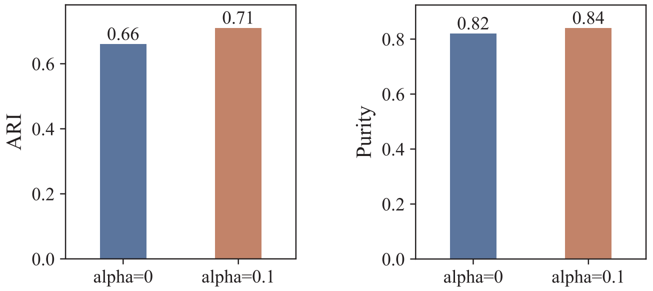

Figure 10: **Ablation on the breast cancer dataset (DCIS identification).** Comparison of SA2E without spatial graph regularization ($\alpha = 0$) and the default setting ($\alpha = 0.1$) in terms of **ARI** (left) and **purity** (right) for identifying spots within the DCIS region. Removing the spatial graph term reduces ARI from 0.71 to 0.66 and purity from 0.84 to 0.82.

tion performance. As shown in Fig. 11, enabling the spatial constraint improves ARI and purity.

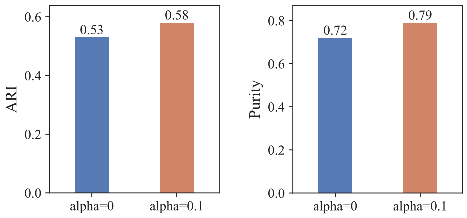

Figure 11: **Ablation on the MOB dataset (spatial domain identification).** Comparison of SA2E without spatial graph regularization ($\alpha = 0$) and the default setting ($\alpha = 0.1$) in terms of **ARI** (left) and **purity** (right). Enabling the spatial graph term improves ARI from 0.53 to 0.58 and purity from 0.72 to 0.79.

S5.2 Interaction between α and data characteristics on the simulated dataset

We assess how the effect of α varies under different data characteristics on the simulated ST dataset. We use the total number of cell types as an indicator of tissue complexity and the number of cells per spot as an indicator of spot density. As shown in Fig. 12, SA2E generally performs best under a small α, with peak performance around $\alpha \approx 0.1$, whereas overly large α leads to performance degradation. PCC differences across complexity settings are modest (panel A), while PCC curves across spot-density settings are nearly indistinguishable in the small-α regime (panel B). Beyond a few dominant cell types, many additional types have negligible proportions in most spots, which increases the number of

near-zero entries and makes the overall PCC less sensitive.

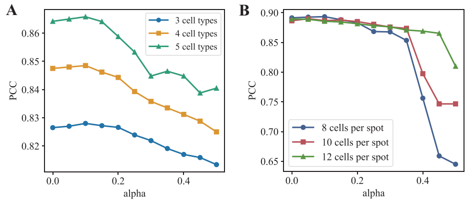

Figure 12: **Interaction between spatial graph strength α and data characteristics on the simulated ST dataset.** PCC of SA2E under different values of α across varying simulation settings. **(A)** Tissue complexity is varied by changing the number of cell types (3, 4, and 5). **(B)** Spot density is varied by changing the number of cells per spot (8, 10, and 12). The results show that the effect of spatial regularization depends on data characteristics, and overly large α can lead to performance degradation.

S5.3 Effect of alternative spatial graph constructions

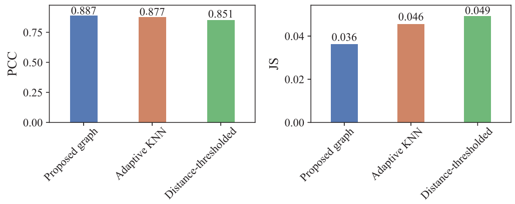

Figure 13: **Comparison of alternative spatial graph constructions on the simulated ST dataset.** Performance of SA2E under three graph construction strategies, including the proposed KNN graph, an adaptive KNN graph, and a distance-thresholded graph. Deconvolution accuracy is evaluated by PCC (left) and JS divergence (right), where higher PCC and lower JS indicate better performance.

We further evaluate whether different spatial graph constructions lead to different performance outcomes. We compare the proposed graph construction with two alternatives, including an adaptive KNN graph [1] and a distance-thresholded graph. As shown in Fig. 13, different constructions yield measurable performance differences, and the proposed graph achieves the best overall performance in this comparison.

References

[1] Yongda Cai, Joshua Zhexue Huang, and Jianfei Yin. A new method to build the adaptive k-nearest neighbors similarity graph matrix for spectral clustering. *Neurocomputing*, 493:191–203, 2022.
